# DCC-YOLOv8n: a lightweight model for maize seedling and weed recognition in complex farmland environments

**DOI:** 10.3389/fpls.2026.1901787

**Published:** 2026-07-29

**Authors:** Jiapeng Cui, Shengqiang Hao, Yinyin Yang, Zhuo Chen, Nan Bai

**Affiliations:** 1 College of Mechanical Engineering, Chongqing Sanxia University of Science and Technology, Chongqing, China; 2 Chongqing Key Laboratory of Light Mountain Intelligent Agricultural Machinery, Chongqing Sanxia University of Science and Technology, Chongqing, China; 3 College of Information Engineering, Liaoning Vocational University of Technology, Jinzhou, China

**Keywords:** field weeds, Jetson Nano, object detection, recognition system, smart agriculture

## Abstract

**Introduction:**

To achieve high-precision and high-efficiency recognition of maize seedlings and weeds in complex field environments while meeting the deployment requirements of resource-constrained edge devices, this study constructed a lightweight object detection model, DCC-YOLOv8n.

**Methods:**

Based on YOLOv8n, the model incorporates three key improvements: a dynamic convolution module to enhance feature diversity, a context-guided module to improve target identification in complex backgrounds, and a content-aware reassembly of features (CARAFE) module to improve small-object detection. A self-built dataset of maize seedlings and weeds containing 1,200 images was utilized, incorporating multidimensional data augmentation.

**Results:**

Experimental results demonstrated that DCC-YOLOv8n achieved a precision of 90.1% and a recall of 95.5%, with mAP@0.5 and mAP@0.5:0.95 reaching 93.7% and 73.9%, respectively, outperforming YOLOv8n and mainstream comparison models, including Faster R-CNN and YOLOv5n. Deployment on the NVIDIA Jetson Nano edge-computing platform achieved a real-time inference speed of 18.6 FPS with mAP@0.5 of 90.8%, verifying the feasibility of its application and deployment on actual farmland edge devices.

**Discussion:**

The proposed DCC-YOLOv8n model achieved an optimal balance between accuracy, lightweight architecture, and real-time performance. The constructed field recognition system effectively addresses the challenges of complex farmland scenarios and provides a viable technical solution for intelligent operations in precision agriculture.

## 1 Introduction

The continuous surge in the global population has rendered food security a severe challenge that requires urgent resolution (Shehu et al., 2025). As a critical global staple crop, maize has an irreplaceable strategic position in maintaining the stability of the global agricultural production system and food supply.Its significance lies not only in securing basic food supplies but also in acting as a vital engine for maintaining social stability, stimulating agricultural economic vitality, and improving the livelihoods of rural residents. However, weed stress poses a serious threat to crop production (Zhang et al., 2023), and weeds are estimated to cause global crop yield losses of up to 43% annually (Chen et al., 2022). Traditional weed management relies primarily on chemical herbicides (Li et al., 2021), which, despite playing an important role in protecting crop health and increasing yields (Elstone et al., 2020), face significant drawbacks under the current extensive whole-field broadcast spraying mode: herbicides are overapplied even in weed-free areas. This practice negatively affects farmland ecosystems and substantially increases agricultural operating costs (Etienne et al., 2021; Zhao et al., 2022). Therefore, the development of automated crop and weed detection systems to achieve precise weed identification and intelligent spraying is crucial for advancing green and sustainable agricultural development and enhancing global food productivity.

With the rapid evolution of computer vision and artificial intelligence, machine vision technology (Da Silva Ferreira et al., 2024; Ding et al., 2024; Dono et al., 2024) has been deeply applied to various stages of agricultural production, demonstrating immense potential, particularly in seedling-stage weed recognition tasks. The introduction of deep learning technology enables agricultural equipment to decouple crop and weed features precisely, providing core technical support for farmland mechanization, intelligent weeding, and variable-rate operations (Li et al., 2022; Xiang et al., 2024). In early agricultural applications, traditional machine learning methods mainly included algorithms such as multivariate data analysis (Gonzalez-Gonzalez et al., 2021), principal component analysis (PCA) (Rahman et al., 2020), template matching (Daga and Garibaldi, 2020), and random forest (Palumbo et al., 2021). These methods rely heavily on handcrafted features, such as color, texture, and morphology of crops and weeds in images, for classification. However, in complex and variable unstructured farmland environments, the feature expression capabilities of such methods are limited, resulting in low generalization performance and recognition accuracy, making it difficult to meet the operational requirements of modern precision agriculture. In recent years, convolutional neural networks (CNNs), pioneered by LeCun et al. (1989), have risen rapidly and been widely applied to tasks such as image segmentation, disease detection, and weed recognition in the field of agronomy. For instance, Olsen et al. (2019) constructed the DeepWeeds dataset for multiclass weeds and trained InceptionV3 and ResNet-50 recognition models based on it; Ahmad et al. (2021) evaluated the performance of three mainstream CNN models, with results showing that the VGG-16 model achieved optimal accuracy; Herrera et al. (2014) proposed a method to distinguish between monocotyledonous and dicotyledonous weeds, achieving a recognition accuracy of 85.8%; and Peteinatos et al. (2020) developed a weed recognition framework based on CNNs, where the improved model’s mean average precision (mAP) exceeded 74%.

Recently, various advanced deep learning-based object detection frameworks, such as Fast R-CNN (Sun et al., 2025), Faster R-CNN (Yang et al., 2022), Cascade R-CNN (Cai and Vasconcelos, 2018), RetinaNet (Ale et al., 2018), RTMDet (Lyu et al., 2022), and the YOLO series (Badgujar et al., 2024) models, have been successfully applied to crop and weed recognition tasks. To address the challenge of seedling and weed recognition, some studies (Wang et al., 2026) constructed new detection models by integrating the ResNeXt backbone with the faster R-CNN algorithm, verifying superior accuracy on specific datasets. Zhang et al (Zhang et al., 2023). introduced the CBAM attention mechanism into the faster R-CNN architecture and utilized VGG-19 as the backbone, achieving a mean detection accuracy as high as 99.16%. Peng et al. (2022) used an improved RetinaNet model to detect weeds in paddy fields, significantly enhancing model robustness under complex lighting and occlusion environments. Mu et al. (2022) combined ResNeXt with FPN to build an improved model, which also achieved a recognition accuracy exceeding 95%. Although these two-stage or complex one-stage models possess high detection accuracy, they are typically accompanied by massive parameter counts and long inference delays, which make them difficult to adapt to terminal operation scenarios in the field.

By contrast, the YOLO series models have received extensive attention in the agricultural sector because of their excellent balance between detection speed and accuracy. The YOLO algorithm treats object detection as a regression problem, directly outputting target bounding boxes and category probabilities in an end-to-end manner, offering significant advantages, such as fast inference speed and low computational overhead. Quan et al. (2022) proposed a mobile weeding robot platform integrating the YOLOv3 model, and field trials showed that the system achieved a weed clearance rate of 85.91%, with a crop damage rate of only 1.17%. Jiang et al. (2023) developed an intra-row mechanical weeding system based on an improved YOLOv5 with a visual model detection accuracy of 95%; at an agricultural machinery operating speed of 3.28 km/h, the intra-row weed clearance accuracy remained at 80.25%. Regarding the requirement for lightweight edge-side deployment, Hu et al. (2024) improved the YOLOv7 architecture and proposed a multimodule-YOLOv7-L model, achieving a plant recognition accuracy of 97.5%. Fan et al. (2024) proposed a lightweight YOLOv5 model that reduced the parameters by 82.3% and compressed the model volume by 91.6%, while decreasing the inference time by 57.14% and increasing the mAP by 9.1%. Furthermore, Wu et al. (2023) introduced Res2block, dilated convolutions, and multiscale feature fusion modules based on YOLOv4, which significantly enhanced the recognition capability for dense small targets. Chen et al (Chen et al., 2022). improved YOLOv4 for sesame field weeds, achieving F1-scores of 0.91 and 0.92, an mAP of 96.16%, and a detection frame rate (FPS) of 36.8. Wang et al. (2024) constructed the CSCW-YOLOv7 model to recognize five weed species in complex wheat fields, achieving precision, recall, and mAP of 97.7%, 98%, and 94.4%, respectively, while effectively reducing the number of parameters and computational complexity (FLOPs). Gómez et al (Gómez et al., 2025). assessed Faster R-CNN, RT-DETR, and YOLOv11 for weed detection in intra- and inter-row zones. YOLOv11 emerged as the top performer with a mAP of 97.5% and 34 FPS, meeting real-time requirements. Hardware deployment yielded a mAP of 94.4%, supporting the viability of YOLO models for early weed detection with minimal crop disturbance. Shi et al. (2025) developed a modified detector named YOLO11-CGB for Chinese cabbage seedling detection. By replacing standard convolutions with the more efficient GhostConv, integrating a simplified Bidirectional Feature Pyramid Network (BiFPN) into the neck, and embedding a Convolutional Block Attention Module (CBAM) into the backbone, the model achieved a precision of 94.7%, a recall of 93.0%, and a mAP of 97.0%. This architecture significantly mitigated false negatives and false positives. Li et al. (2026) proposed RCW-YOLOv10, an indirect weed detection framework based on YOLOv10. By incorporating a Receptive Field Dilation (RFD) module and a C2f-WDBB module, the model enhances feature preservation and multi-scale fusion efficiency. Compared with the baseline YOLOv10, RCW-YOLOv10 reduces the parameter count by 1.04 million while significantly improving detection performance, achieving increases of 3.5, 1.5, and 1.1 percentage points in Precision, Recall, and mAP50, respectively, under field conditions. This approach streamlines the weed identification process in tested bok choy fields.

While the YOLO series algorithms demonstrate remarkable performance in generic object detection, their direct application to unstructured, high-density field environments encounters several critical bottlenecks. Firstly, lightweight variants (e.g., YOLOv8n), constrained by limited network depth and width, often suffer from insufficient feature discriminability when extracting maize seedlings and coexisting weeds—targets characterized by high visual similarity in color and texture—leading to frequent misclassification (Varghese and Sambath, 2024). Secondly, the conventional SPPF module in standard YOLO architectures relies solely on max-pooling to capture multi-scale contexts, lacking explicit modeling of local-to-global semantic information, which impedes the differentiation of morphologically similar crops against complex backgrounds. Thirdly, the default nearest-neighbor upsampling (Upsample) fails to account for feature map content, frequently resulting in the loss of fine-grained details for tiny weed targets during the decoding process. Addressing these challenges, recent advances in agricultural computer vision have demonstrated that incorporating dynamic convolution to enhance nonlinear feature representation (Chen et al., 2025), adopting context-guided blocks to fuse multi-scale semantic information (Mahmudul et al., 2021), and utilizing Content-Aware ReAssembly of FEatures (CARAFE) to improve small-object reconstruction (Zhang et al., 2023) can effectively boost detection robustness in complex agricultural scenarios. Motivated by these findings, this study proposes DCC-YOLOv8n, a lightweight detection model based on the YOLOv8n baseline. By integrating Dynamic Convolution (DC), the Context Guided Block (CG), and CARAFE, the proposed architecture aims to strike an optimal balance between model compactness and detection accuracy, fulfilling the stringent requirements for real-time field recognition on resource-constrained edge devices. The principal contributions of this work are summarized as follows:

① Model Design: A lightweight maize seedling and weed recognition model, DCC-YOLOv8n, oriented toward complex farmland environments, was constructed. This model is suitable for field recognition tasks under resource-constrained conditions and extends the application of the YOLOv8 model in the agricultural domain.
② Robustness in Unstructured Environments: Data augmentation techniques were employed to simulate various meteorological conditions, effectively enhancing the model’s recognition robustness in complex farmland environments, with performance verified through experiments.
③ Implementation of the Recognition System: The model was successfully deployed on a Jetson Nano edge-computing device, achieving real-time field recognition in offline environments. The system provides core technical support for precision weeding and variable-rate agricultural machinery operations.

## 2 Materials and methods

### 2.1 Self-built dataset

#### 2.1.1 Data acquisition

The maize seedling and weed image dataset used in this study were collected between May 15 and May 30, 2025, at the Agricultural Science and Technology Park of Heilongjiang Bayi Agricultural University and campus experimental fields in Anda City, Heilongjiang Province. The images were captured using a smartphone, and the relevant hardware parameters are listed in **Table 1**. To ensure data validity and robustness, the acquisition conditions were controlled across multiple dimensions: (1) the target area was required to have a complete subject and clear edges; (2) the shooting time was restricted to 5:00–19:00 daily; (3) the shooting distance was maintained within the range of 0.30 m to 0.50 m; and (4) the shooting tilt angle was controlled within approximately ±30°. Using these diverse acquisition strategies, a dataset characterized by image diversity and background complexity was constructed. A total of 2,000 raw images were collected, saved in.jpg format, and standardized to 640 × 640 pixel RGB images using Adobe Photoshop 2020 (version 21.0.1; Adobe Systems Incorporated, San Jose, CA, USA), thereby providing the data foundation for subsequent model training and testing.

**Table 1:** Parameter table of data acquisition equipment.

Camera parameters	Huawei Mate 50	Honor Magic 5	Xiaomi 12 S	iPhone 14
Pixel	50 million	50 million	50 million	48 million
Resolution	8192 × 6144	8192 × 6144	8192 × 6144	8192 × 6144
Aperture	f/1.4	f/1.6	f/1.9	f/1.78
Sensor model	IMX 766	IMX 700	IMX 707	IMX 703

#### 2.1.2 Dataset construction

**Figure 1:**
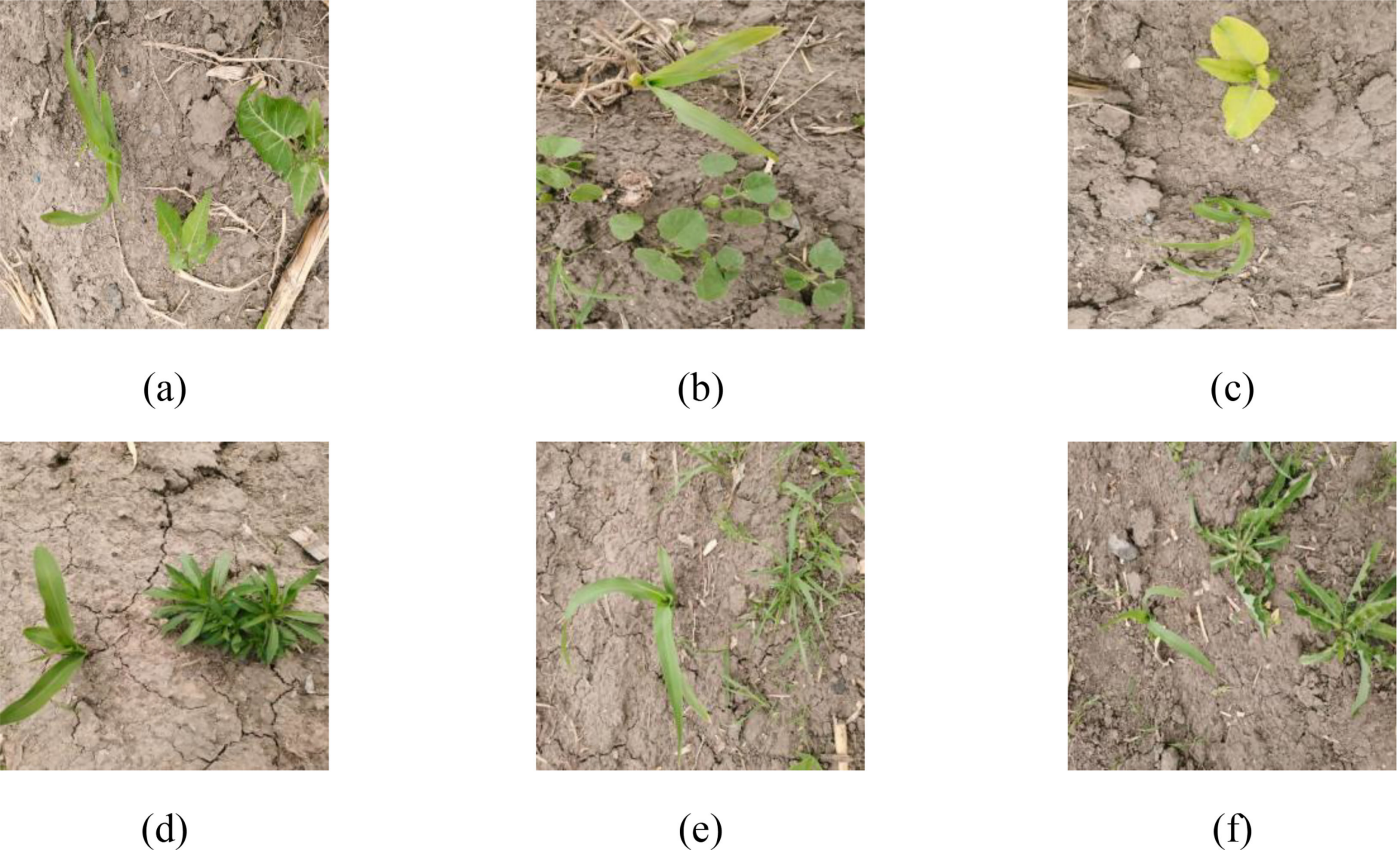
Images of maize seedlings and weeds. **(a–c)** Weeds exhibit obvious differences in color and morphology compared with maize seedlings, making them relatively easy to distinguish. **(d–f)** In contrast, weeds are highly similar to maize seedlings in terms of color and leaf morphology, which significantly increases the difficulty of recognition.

To eliminate image quality inconsistencies caused by human or equipment factors, a systematic data cleaning process was performed, including (1)manual pre-screening was conducted to remove non-field-of-view images, globally defocused images, and corrupted or undecodable files; (2) screening for high-similarity images using a pixel contrast algorithm, followed by manual re-examination for deduplication; and (3) with the assistance of agricultural experts, 1,200 qualified images containing both maize seedlings and weeds were ultimately selected to form the standard dataset for model training and testing. Typical image examples are shown in **Figure 1**.

**Figure 2:**
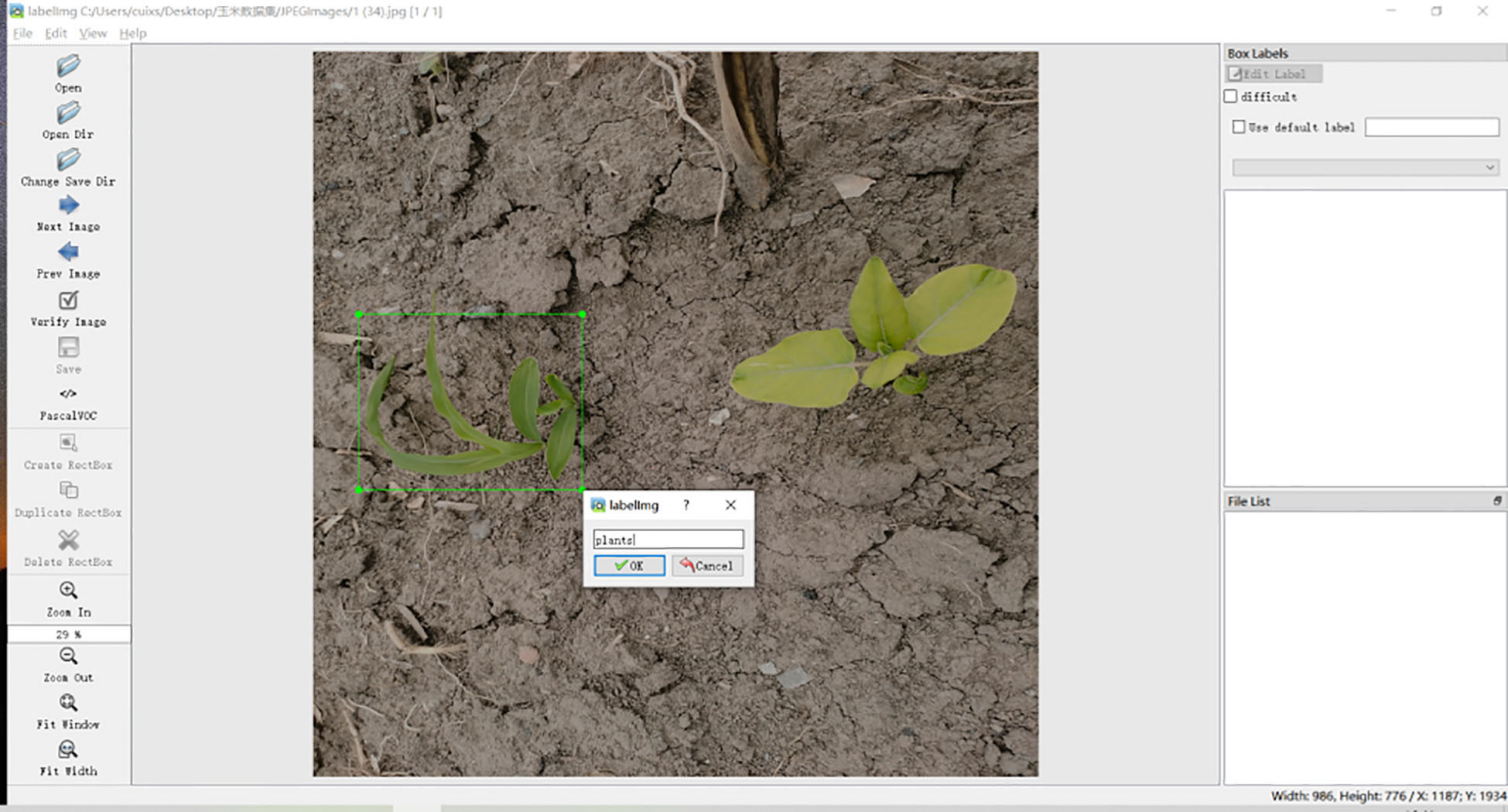
Interface of the LabelImg annotation tool. During the annotation process, rectangular bounding boxes were used to label the targets in the images. Maize seedlings were labeled as “plants” while weeds were labeled as “grass”.

The LabelImg application was used to annotate the images for object detection. Maize seedlings were uniformly labeled as “plants,” and associated weeds were labeled as “grass.” Upon completion of annotation, the dataset was organized into the YOLO format and named the Plant and Grass Detection (PGD) dataset. The LabelImg software interface is shown in **Figure 2**.

To ensure balanced data distribution and experimental reproducibility, the PGD dataset was divided into training, validation, and test sets in a ratio of 60:20:20 (Cui et al., 2024). The validation set remained invisible during the model training process, adhering to the standard three-stage data-partitioning specifications. The partitions of the PGD training, validation, and test sets are listed in **Table 2**.

**Table 2:** Division of training set, validation set, and test set for the PGD dataset.

Dataset category	Image size (pixels)	Division proportion (%)	Number of samples (pieces)	Total sample size (samples)
Training set	640 × 640	60.00	720	1200
Test set		20.00	240	
Validation set		20.00	240	

#### 2.1.3 Data preprocessing

**Figure 3:**
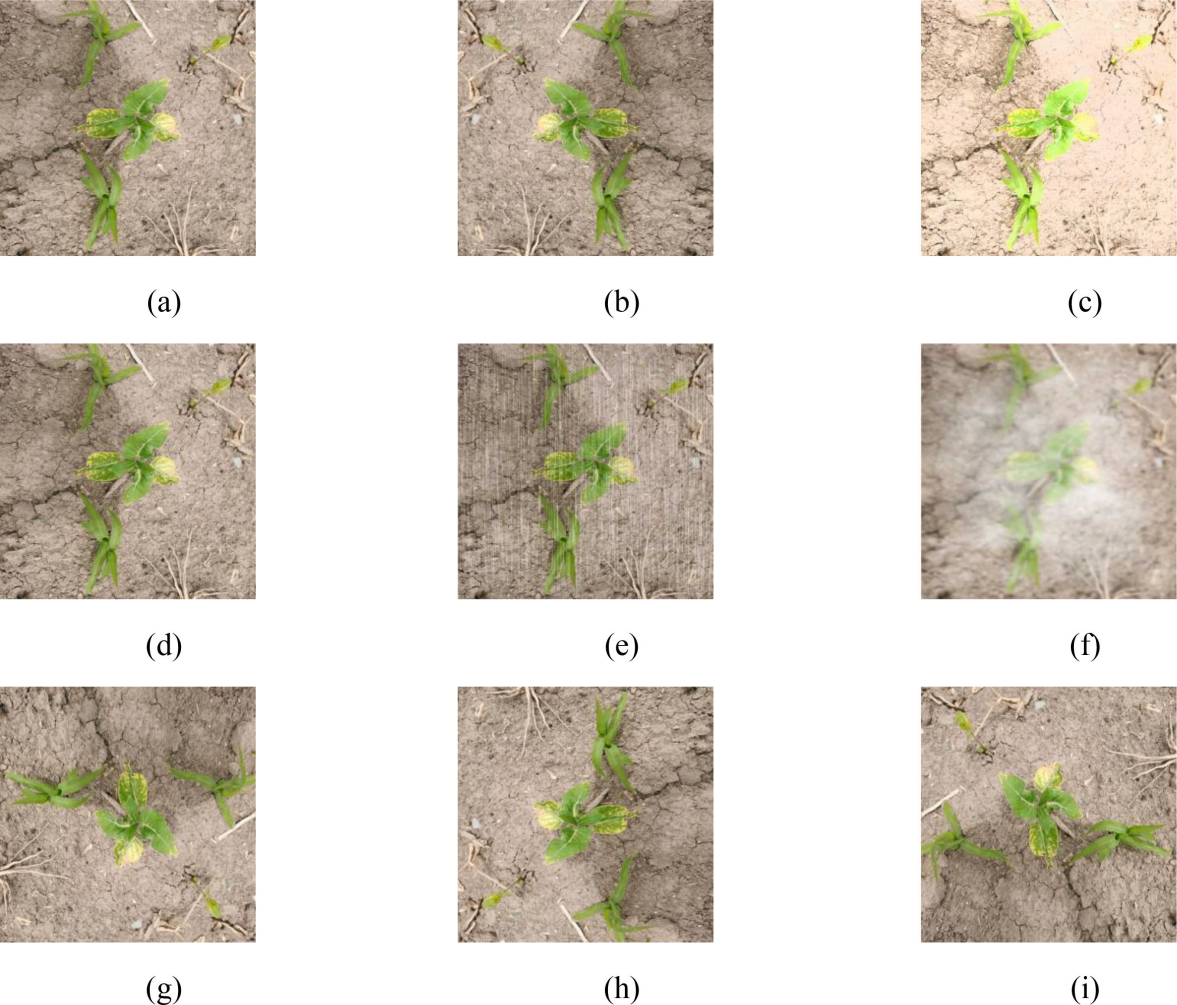
Examples of data augmentation. **(a)** Original image; **(b)** mirroring; **(c)** brightness adjustment; **(d)** Gaussian noise; **(e)** rain simulation; **(f)** fog simulation; **(g)** rotation 90°; **(h)** rotation 180°; and **(i)** rotation 270°.

To alleviate potential overfitting issues caused by the limited scale of the dataset and enhance the adaptability of the model to actual complex field scenarios, a multidimensional data augmentation strategy was employed. This included: ① Angle perturbation: simulating multi-view shooting conditions through random rotation and mirror transformations; ② Illumination perturbation: simulating diverse weather and lighting conditions (e.g., sunny, cloudy, post-rain) by adjusting brightness and contrast; ③ Noise perturbation: simulating abnormal weather conditions such as rain and fog, as well as equipment noise interference, by adding Gaussian noise, salt-and-pepper noise, and simulated fogging effects (Cui and Tan, 2023). Examples of the augmentation effects are shown in **Figure 3**. Specifically, dataset partitioning preceded data augmentation; augmentation was restricted to the training phase; and no image or its transformed variant from the training set appeared in the validation or test sets.

### 2.2 Proposed method

#### 2.2.1 Network model construction

**Figure 4:**
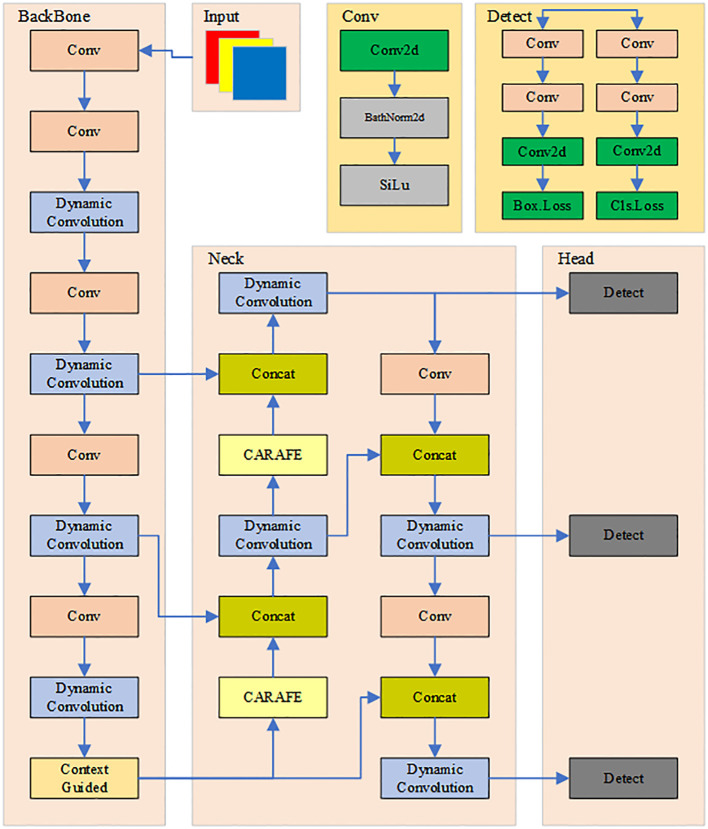
DCC-YOLOv8n network structure diagram. The proposed model introduces three key improvements based on the YOLOv8n architecture: ① The C2f module is replaced with a DC module; ② The SPPF module is replaced with a CG module; ③ The upsample module is replaced with a CARAFE module.

In this study, a lightweight maize seedling and weed detection model, DCC-YOLOv8n, was constructed based on the YOLOv8n architecture. The model incorporates three key improvements over the baseline YOLOv8n: (1) The C2f module was replaced with a dynamic convolution (DC) module (Chen et al., 2020) to enhance the capability of feature diversity extraction, balancing the lightweight design with expressive power. (2) The SPPF module was replaced with a context-guided (CG) block (Tianyi et al., 2020). The ability of the model to identify similar targets in complex backgrounds was improved by extracting and fusing local, surrounding, and global contextual information simultaneously. (3) The upsample module was replaced with the content-aware reassembly of features (CARAFE) module (Wang et al., 2019), enabling adaptive feature reassembly based on image texture and structure. This preserves detail information during feature map upsampling, which is particularly beneficial for detecting tiny weeds. While maintaining high operational efficiency, the model possesses robust pixel-level semantic feature mapping capabilities. The architecture of the DCC-YOLOv8n model is illustrated in **Figure 4**.

##### 2.2.1.1 Baseline model selection

**Figure 5:**
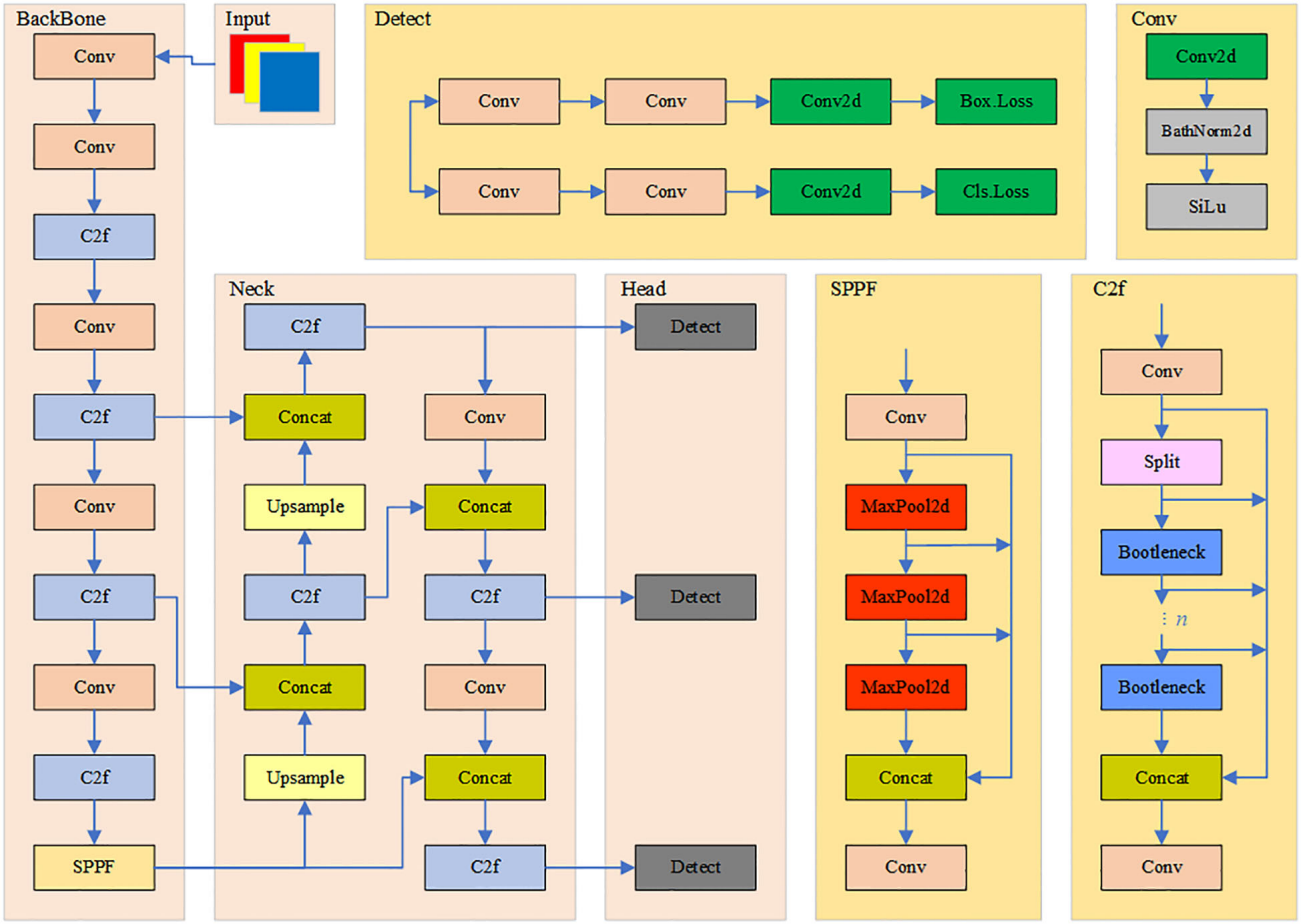
YOLOv8 network structure diagram. The YOLOv8 model consists of four main components: the input layer, backbone network, neck network, and head (detection layer).

YOLOv8 is a state-of-the-art algorithm in the field of object detection released by Ultralytics in January 2023. Building on the structural advantages of the YOLO series, it optimizes the network architecture, training strategies, and loss functions to achieve a superior balance between detection accuracy and inference speed. YOLOv8 offers five versions with varying scales (n, s, m, l, and x) to accommodate diverse computational resources. Among these, YOLOv8n features the smallest model size and the fastest inference speed, making it highly suitable for deployment on resource-constrained embedded devices. Because its lightweight characteristics align closely with the objectives of this study, YOLOv8n was selected as the baseline network architecture. The structure of the YOLOv8n model is shown in **Figure 5**.

##### 2.2.1.2 DC module

**Figure 6:**
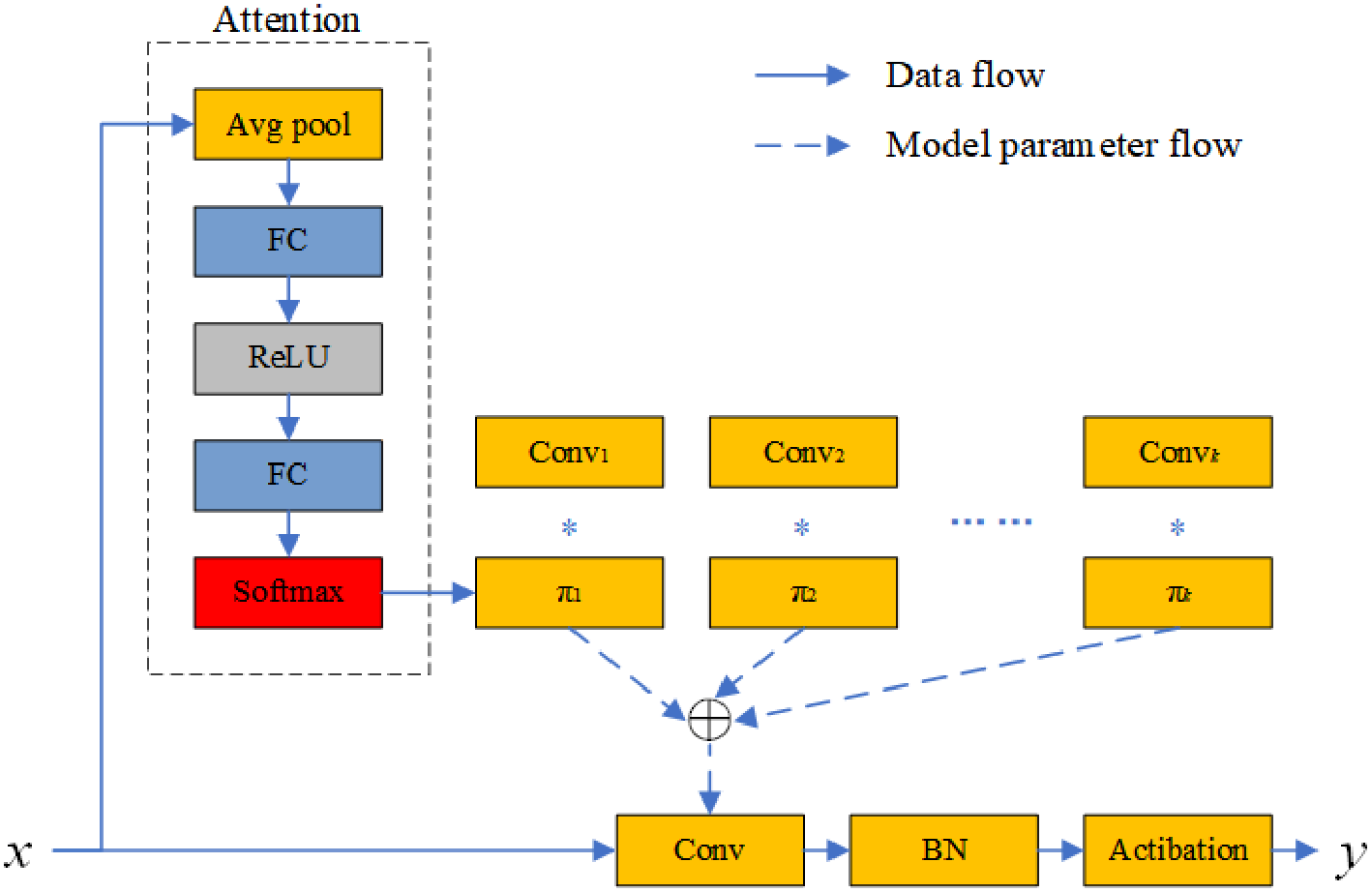
Structure of the DC module. The dynamic convolution module employs *k* convolution kernels with shared size and dimensionality. These kernels are adaptively aggregated using attention weights {π_k_}. By embedding an attention mechanism into a static network structure, the module enables adaptive feature extraction without requiring an additional controller, and supports end-to-end training.

As a lightweight network, YOLOv8n’s limited depth and width can restrict its representation capability. To increase the model complexity without significantly increasing the computational burden, we replaced all original C2f modules with DC modules. By utilizing an input-dependent attention mechanism, this module dynamically aggregates multiple parallel small-scale convolution kernels, thereby enhancing the nonlinear expression of features while maintaining computational efficiency. This improvement helps the model distinguish morphologically similar maize seedlings and weeds in unstructured field environments. To balance computational cost and performance, the hyperparameter was set to k = 6, deviating from the conventional value of 4. This adjustment enhances the model’s adaptability to the morphological diversity of weeds, while the integrated attention mechanism prioritizes high-frequency edge features to suppress complex farmland background textures. The structure of dynamic convolution is shown in **Figure 6**.

##### 2.2.1.3 CARAFE module

**Figure 7:**
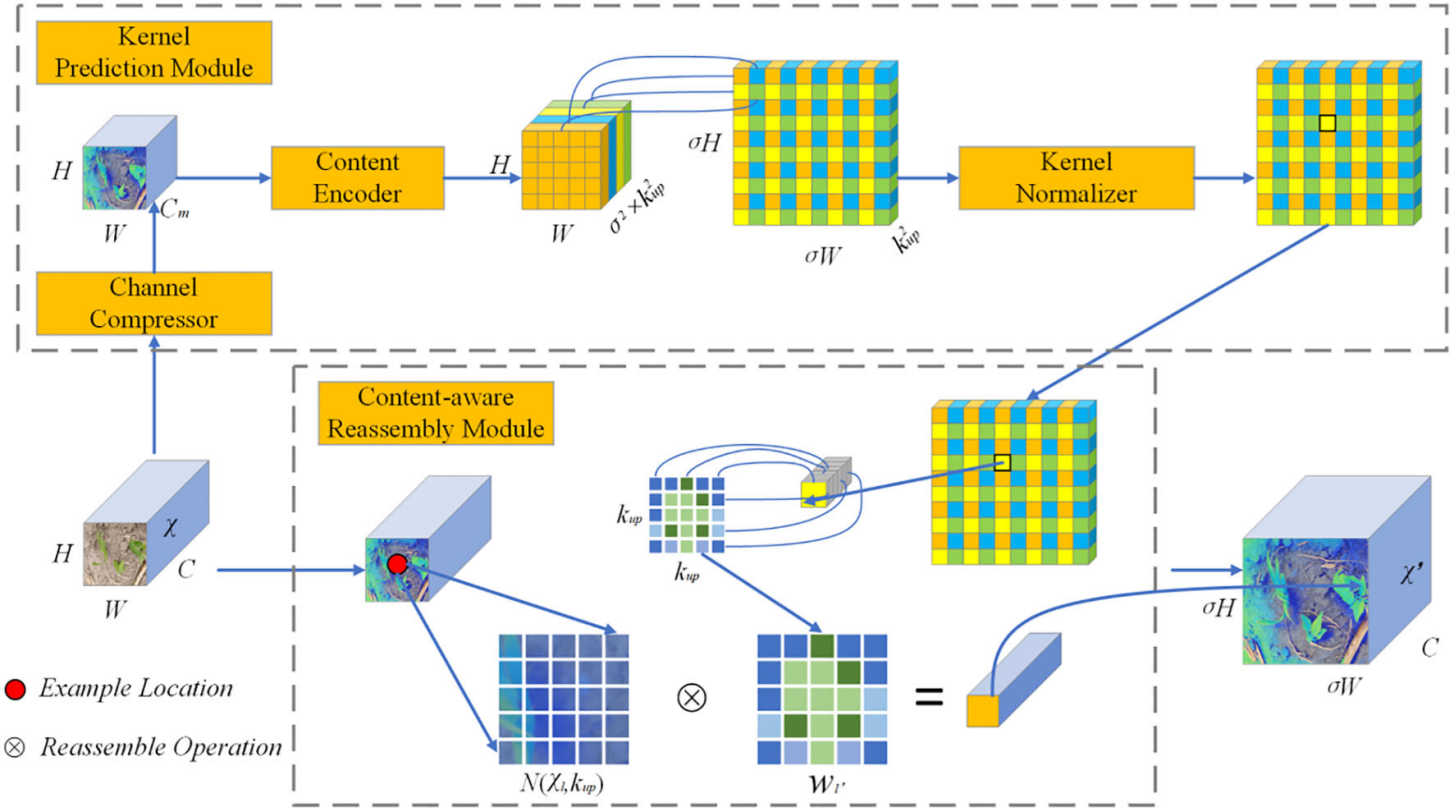
Structure of the CARAFE module. The CARAFE module consists of two core components: the kernel prediction module (KPM) and the content-aware reassembly module (CARM). Based on the underlying content information at each pixel location, this method adaptively predicts reassembly kernels and performs feature reassembly within a predefined local neighborhood.

Feature upsampling is a critical operation in convolutional networks and is essential for dense prediction tasks. The original YOLOv8n uses nearest-neighbor interpolation, which, although computationally efficient, lacks content-aware capability and often leads to loss of details. Consequently, this study adopted the CARAFE module as a replacement. CARAFE offers the following advantages: ① a large receptive field that aggregates broad contextual information; ② content awareness, enabling the dynamic generation of adaptive upsampling kernels based on input features. CARAFE significantly enhances the feature representation of similar targets in unstructured field environments with negligible computational overhead. Specifically, the upsampling kernel size was set to 5×5—larger than the conventional 3×3—to better reconstruct the sparse features of small weeds, while correlating content-aware weights with edge responses to sharpen sensitivity to low-contrast targets. The structure of the CARAFE module is shown in **Figure 7**.

##### 2.2.1.4 CG module

**Figure 8:**
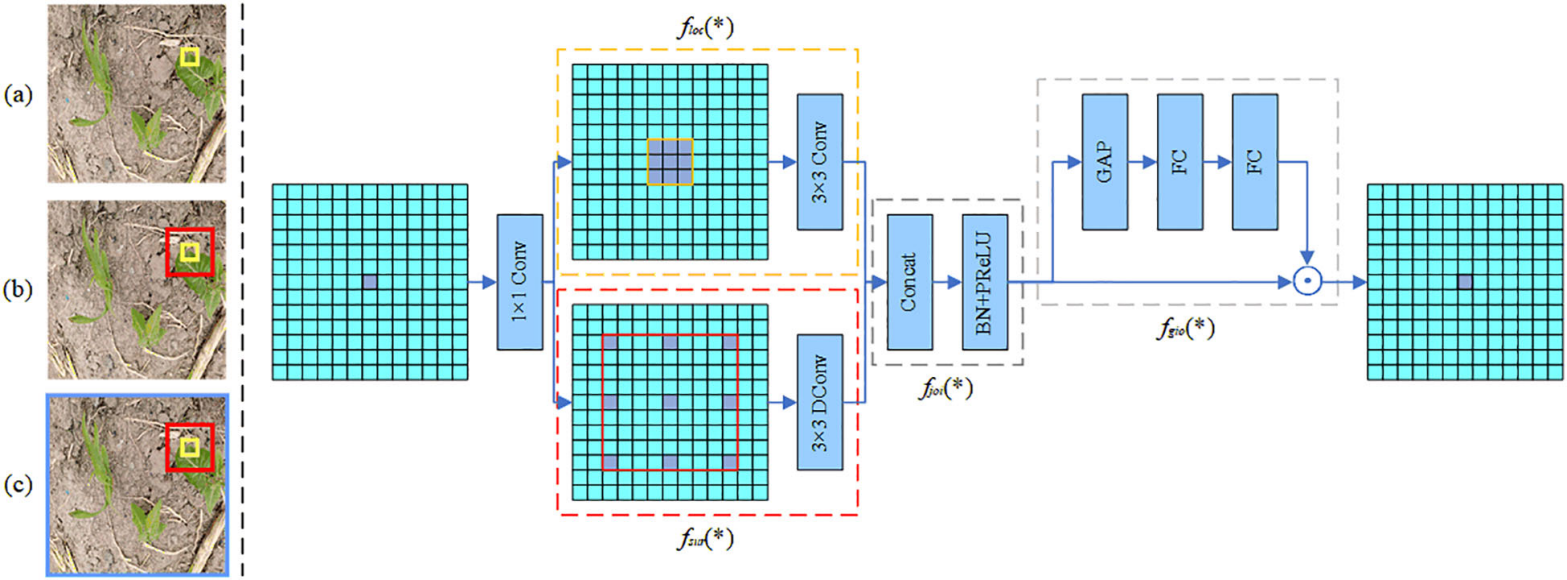
Architecture of the CG module. The CG module is inspired by the mechanism of the human visual system, which relies on contextual information to understand scenes. **(a)** When only the yellow region is observed, its category is difficult to determine. **(b)** The introduction of the surrounding red region as contextual information significantly reduces the ambiguity, indicating that local context plays an important role in semantic understanding. **(c)** When local context (yellow region), surrounding context (red region), and global context (purple region) are jointly considered, the classification confidence can be substantially improved. Therefore, the collaboration of surrounding and global contextual information effectively enhances the model’s discriminative capability in complex scenarios.

To improve the ability of the model to differentiate morphologically similar targets from complex field backgrounds, the original SPPF module was replaced with a CG block. The CG module operates through the synergy of four submodules: a local feature extractor *f_loc_*(*), surrounding context extractor *f_sur_*(*), joint feature extractor *f_joi_*(*), and global context extractor *f_glo_*(*). Initially, *f_loc_*(*) and *f_sur_*(*) extract local features and their surrounding context, respectively, which are then fused into joint features. Subsequently, *f_glo_*(*) extracts the global context to refine the joint features. This module efficiently captures multi-scale contextual information across all network stages with low parametric and memory overhead, significantly enhancing semantic perception in complex scenarios. Specifically, the context aggregation ranges are adjusted to multi-scale kernels(3×3, 5×5, and 7×7) to accommodate scale variations of maize seedlings and weeds across different growth stages. Furthermore, local contrast normalization is introduced to mitigate the adverse effects of uneven illumination. Consequently, the model achieves robust recognition of morphologically similar seedlings and weeds in the field. The network structure of the CG module is illustrated in **Figure 8**.

#### 2.2.2 Real-time deployment verification on edge devices

To verify the deployment potential of the DCC-YOLOv8n model in practical agricultural edge devices, it was deployed on an NVIDIA Jetson Nano development board. The inference speed and resource efficiency were systematically evaluated in a real-world resource-constrained environment. A fair comparison with the baseline YOLOv8n model validated the performance gains of the lightweight improvements for practical applications. The hardware configuration of the NVIDIA Jetson Nano is listed in **Table 3**.

**Table 3:** Hardware configuration parameters of NVIDIA Jetson Nano.

Name	Configuration information
GPU	128-core NVIDIA Maxwell™ architecture GPU
CPU	Quad-core ARM^®^ Cortex^®^-A57 MPCore processor
AI performance	472 GFLOPs
Memory	4 GB 64-bit LPDDR4
Storage	MicroSD (excluding the card)
USB	4 USB3.0 Type-A interfaces, 1 USB2.0 Micro-B interface
Camera	2 x 15-pin 2-channel MIPI CSI-2 camera interfaces
Monitor	1 HDMI 2.0 interface, 1 DP 1.2 interface
Other I/O	40-pin connector (UART, SPI, I2S, I2C, PWM, GPIO)
Specs	100 mm × 79 mm × 30.21 mm
Power supply	5 W/10 W

A standardized workflow was adopted to ensure experimental fairness. The hardware platform was an NVIDIA Jetson Nano, and the software environment was JetPack 5.1.2. Both the official YOLOv8n model and the proposed DCC-YOLOv8n model were exported to the ONNX format and converted into inference engines with FP16 precision using TensorRT 8.5. Both models were evaluated using the same input resolution. Finally, the process was encapsulated as a callable service with an inference warm-up performed at startup to ensure operational stability. The software environment configuration for the NVIDIA Jetson Nano is presented in **Table 4**.

**Table 4:** Software environment configuration for NVIDIA Jetson Nano.

Name	Configuration information
Operating system	Ubuntu 18.04
Programming language	Python 3.6.5, C++
Hardware acceleration library	CUDA 10.2
Computer vision library	OpenCV 4.1.1
Deep learning inference optimizer	TensorRT 8.0.1
Computer vision acceleration library	VPI 1.1.15

**Figure 9:**
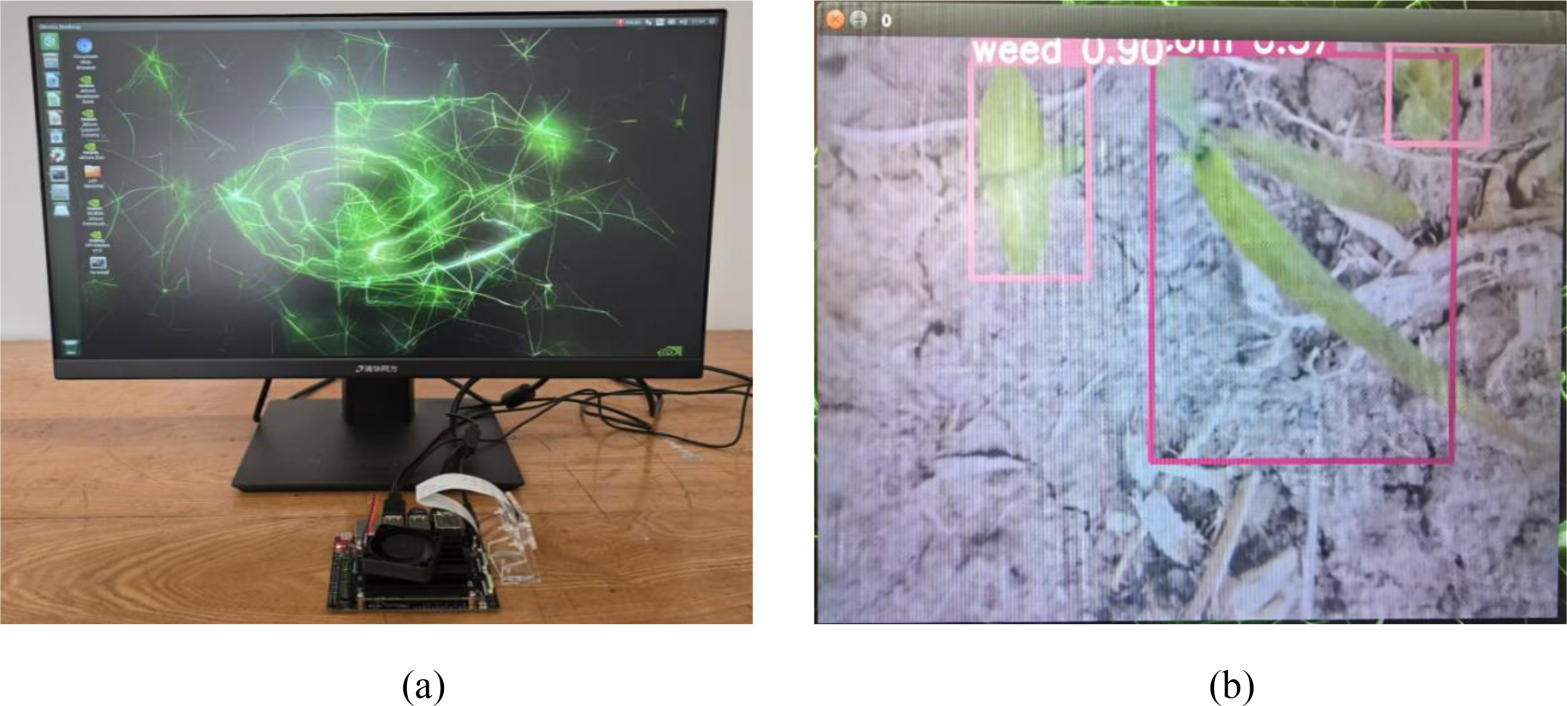
Lightweight maize seedling and weed recognition system for complex farmland environments. **(a)** System framework; **(b)** Visualization interface displaying detected maize seedlings and weeds.

The lightweight maize seedling and weed recognition system oriented toward complex farmland environments is shown in **Figure 9**.

### 2.3 Evaluation metrics

To comprehensively evaluate the model performance, this study adopted precision, recall, mAP, floating point operations (FLOPs), parameters, and frames per second (FPS) as core evaluation metrics across three dimensions: detection accuracy, computational complexity, and inference speed (Gao et al., 2025).

Precision: The proportion of truly positive samples among those predicted as positive by the model.

Recall: The proportion of all actual positive samples correctly predicted by the model.

mAP: The average of the AP values across all categories, serving as the core comprehensive metric for multiclass object detection. mAP@0.5 specifically refers to the mean AP across all categories when the intersection over union (IoU) threshold is set to 0.5. mAP@0.5:0.95 represents the average mAP obtained under multiple IoU thresholds ranging from 0.5 to 0.95 (with a step size of 0.05), providing a more comprehensive reflection of the robustness of the model under different localization accuracy requirements.

FLOPs: The number of floating-point operations required for a single forward inference. This is used to measure the computational complexity of the model.

Parameters: Total number of trainable weights in the model, which is used to measure the model scale.

FPS: The number of images that the model can process per second, which is used to measure the inference speed.

## 3 Results and analysis

### 3.1 Model experiments

#### 3.1.1 Training conditions

##### 3.1.1.1 Hardware configuration

Based on a comprehensive analysis of the experimental content, core requirements for training and optimizing the CNN models, and relevant technical parameters, the hardware configuration for model training was determined by balancing the operational efficiency, quality, and cost. The specific parameters are listed in **Table 5**.

**Table 5:** Hardware configuration parameters.

Name	Configuration information
GPU	NVIDIA GeForce RTX 3060 GPU(12 GB)
CPU	AMD Ryzen 7 6800H(3.2 GHz)
Memory	16 GB
Hard disk	500 GB

##### 3.1.1.2 Software environment

Deep learning frameworks are critical software tools for model research, training, and optimization. Considering the research content of this study and programming environment requirements of the model, the software environment configuration is presented in **Table 6**.

**Table 6:** Software environment configuration.

Name	Configuration information
Operating system	Ubuntu 20.04
Programming language	Python 3.8.5
Deep learning framework	PyTorch 1.8.1
Scientific computing	NumPy, SciPy, Scikit-learn, Pandas, Seaborn, Matplotlib
Computer vision library	OpenCV 4.8.0
Hardware acceleration	CUDA 11.8, CUDNN 8.9.2
Development environment	PyCharm Professional 2021.2

#### 3.1.2 Training parameters

Considering the characteristics of the dataset, network architecture, task type, hardware resources, and prior experience, the training parameters for the DCC-YOLOv8n model were established. The specific settings are listed in **Table 7**.

**Table 7:** Training parameter settings for the DCC-YOLOv8n model.

Name	Configuration information
Imagesize	640 × 640
Epoch	100
BatchSize	16
Optimizer	SGD
Loss Function	Default Loss Function of YOLOv8n
Learning rate	0.01
Schedule	Cosine annealing decay
Momentum	0.937
Weight decay	0.0005

#### 3.1.3 Training strategy

To ensure fair comparisons and eliminate biases introduced by prior knowledge, all experimental models were trained entirely from scratch on the self-built PGD dataset. Although official pre-trained weights derived from large-scale datasets are available for the comparative models, these were deliberately not utilized in this study. Instead, all network parameters were randomly initialized to neutralize the performance advantages conferred by large-scale pre-training, thereby ensuring that observed performance differences stem solely from algorithmic improvements rather than discrepancies in data distribution or prior knowledge.

### 3.2 Results analysis

#### 3.2.1 Comparative analysis of model experimental results

##### 3.2.1.1 Ablation study

**Figure 10:**
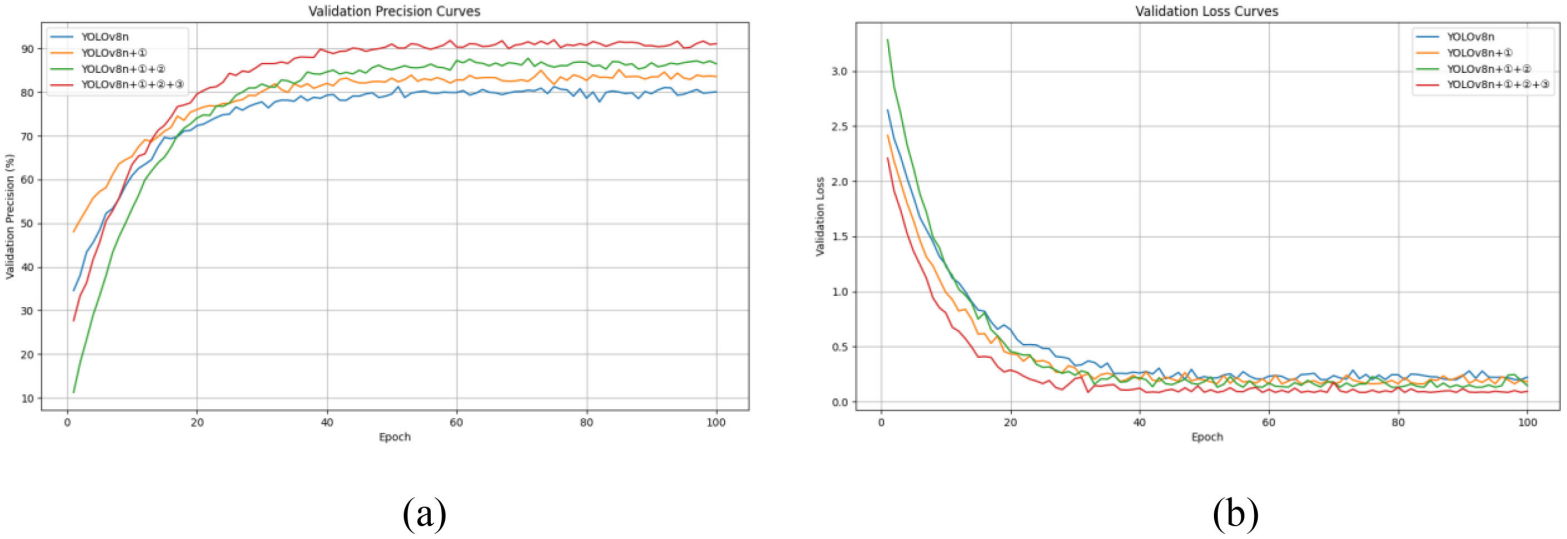
Loss and accuracy curves of different models. **(a)** Loss curves; **(b)** accuracy curves.

A progressive ablation study was designed using the original YOLOv8n as the baseline to systematically verify the contribution of each improved module to model performance. The settings were as follows: ① replacing the C2f module with the DC module; ② replacing the SPPF module with the CG block; ③ replacing the upsample module with the CARAFE module. The experimental results are presented in **Table 8**. The accuracy and loss curves for each model are shown in **Figure 10**.

**Table 8:** Ablation experiment results.

Model	Precision (%)	Recall (%)	mAP@0.5 (%)	mAP@0.5–0.95 (%)	FLOPs (G)	Parameters (M)	FPS
YOLOv8n +①	83.4 ± 0.3	91.7 ± 0.3	89.2 ± 0.3	58.1 ± 0.3	8.8	3.4	54.5
YOLOv8n +①+②	86.3 ± 0.3	94.0 ± 0.3	92.1 ± 0.2	67.3 ± 0.3	10.1	3.8	46.0
YOLOv8n +①+②+③	90.1 ± 0.3	95.5 ± 0.3	93.7 ± 0.2	73.9 ± 0.3	11.2	4.0	41.3
YOLOv8n	79.5 ± 0.4	84.4 ± 0.3	83.2 ± 0.3	51.3 ± 0.3	8.1	3.1	58.2

All results are reported as mean ± standard deviation over five independent training runs with different random seeds. Deterministic metrics (FLOPs, Parameters, FPS) are excluded from variance reporting.

From the ablation results in **Table 8**, the following observations can be made. Compared with the baseline YOLOv8n, the YOLOv8n + DC model enhanced the nonlinear expression while maintaining feature extraction capabilities by replacing the original C2f module with the dynamic convolution module. This improved the representation of the target features in complex farmland backgrounds. Consequently, mAP@0.5 increased by 6.0%, and mAP@0.5:0.95 increased by 6.8%. Regarding the operational efficiency, the FPS decreased slightly to 54.5 (a 6.4% reduction from the baseline). The computational complexity (FLOPs) increased to 8.8 G (an 8.6% increase), and the number of parameters reached 3.4 M (a 9.7% increase).

The YOLOv8n + DC + CG model, compared with the model using only the DC module, further replaced the SPPF module with a CG block. This module parallelly extracts and fuses local, surrounding, and global contextual information, thereby significantly enhancing the target discrimination capability of the model in complex backgrounds, at the cost of additional computational overhead. Accordingly, mAP@0.5 and mAP@0.5:0.95 increased by 2.9% and 9.2%, respectively. The FPS dropped to 46.0 (a 15.6% decrease from the previous stage), while FLOPs rose to 10.1 G (a 14.8% increase) and the parameters reached 3.8 M (an 11.8% increase).

The DCC-YOLOv8n model (integrating all three improvements), compared with the version with only the first two modules, replaced the upsample module with the CARAFE module. This module adaptively reassembles upsampling kernels based on feature content, better preserves detailed information, and is particularly advantageous for small weed targets with similar morphologies, although it further increases the computational burden. Consequently, mAP@0.5 significantly improved by 1.6%, and mAP@0.5:0.95 increased by 6.6%. The FPS decreased to 41.3 (a 10.2% reduction), while FLOPs increased to 11.2 G (a 10.9% increase), and parameters reached 4.0 M (a 5.3% increase).

The ablation study demonstrates that all improvement schemes for the original YOLOv8n effectively enhanced the detection performance for maize seedlings and weeds in complex farmland environments. Compared with the baseline YOLOv8n, the final DCC-YOLOv8n achieved a 10.5% increase in mAP@0.5 and 22.6% increase in mAP@0.5:0.95. While FLOPs and parameters increased by 38.3% and 29.0%, respectively, and the FPS decreased by 29.0%, the results indicate that the proposed modules work synergistically to significantly bolster the detection accuracy and robustness at a reasonable cost of model complexity and inference speed.

##### 3.2.1.2 Comparative analysis with other models

**Figure 11:**
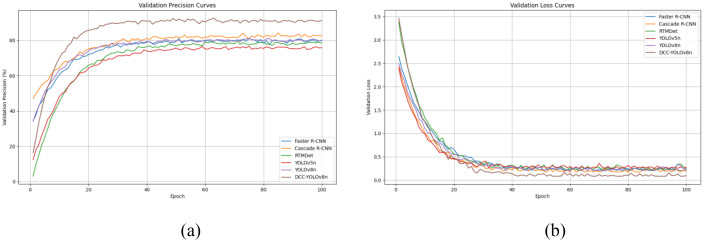
Loss and accuracy curves of different models. **(a)** Loss curves; **(b)** accuracy curves.

**Figure 12:**
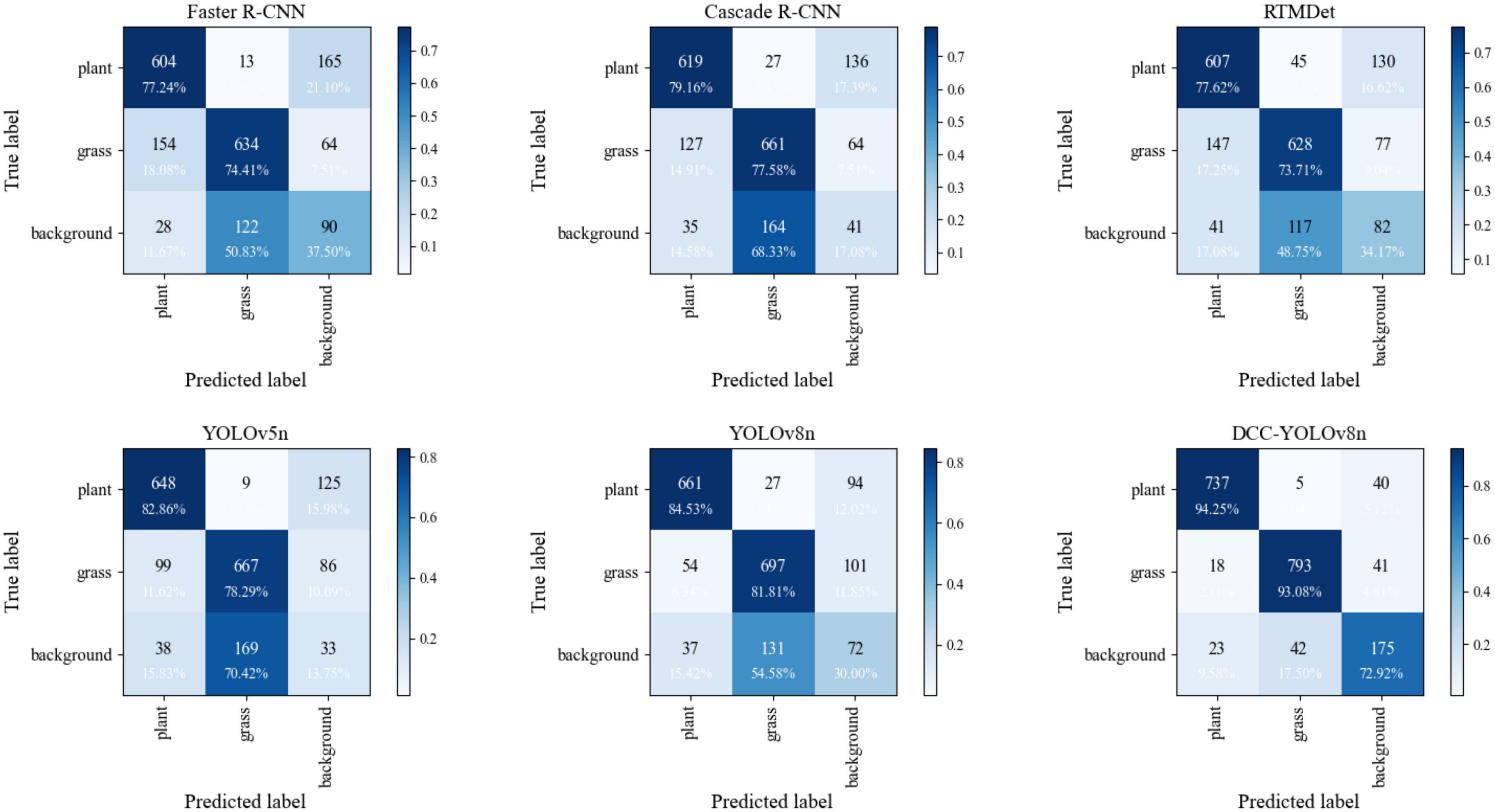
Confusion matrices of compared models.

To objectively evaluate the comprehensive performance of DCC-YOLOv8n, a quantitative comparison was conducted with several mainstream object-detection algorithms, including two-stage detectors (Faster R-CNN and Cascade R-CNN) and one-stage detectors (RTMDet, YOLOv5n, and baseline YOLOv8n). The comparison results are summarized in **Table 9**. The accuracy and loss curves for each model are shown in **Figure 11**. The confusion matrices of the compared models are presented in **Figure 12**.

**Table 9:** Comparison test results.

Model	AP@0.5 plant (%)	AP@0.5 grass (%)	Precision (%)	Recall (%)	mAP@0.5 (%)	mAP@0.5–0.95 (%)	FLOPs (G)	Parameters (M)	FPS
Faster R-CNN	77.2 ± 0.4	74.4 ± 0.4	76.2 ± 0.4	79.5 ± 0.3	75.8 ± 0.4	45.5 ± 0.3	95.2	41.3	19.1
Cascade R-CNN	79.2 ± 0.3	77.6 ± 0.3	79.0 ± 0.3	82.4 ± 0.4	78.4 ± 0.3	49.7 ± 0.3	67.4	69.2	15.3
RTMDet	77.6 ± 0.5	73.7 ± 0.5	75.5 ± 0.5	78.1 ± 0.4	75.7 ± 0.5	46.1 ± 0.4	14.8	3.2	58.7
YOLOv5n	82.9 ± 0.3	78.3 ± 0.3	79.3 ± 0.4	81.9 ± 0.3	80.6 ± 0.3	50.8 ± 0.4	7.1	1.9	62.3
YOLOv8n	84.5 ± 0.3	81.8 ± 0.3	79.5 ± 0.4	84.4 ± 0.3	83.2 ± 0.3	51.3 ± 0.3	8.1	3.1	58.2
DCC-YOLOv8n	94.3 ± 0.2	93.1 ± 0.2	90.1 ± 0.3	95.5 ± 0.3	93.7 ± 0.2	73.9 ± 0.3	11.2	4.0	41.3

All results are reported as mean ± standard deviation over five independent training runs with different random seeds. Deterministic metrics (FLOPs, Parameters, FPS) are excluded from variance reporting.

The comparative experimental results presented in **Table 9** indicate that the two-stage detectors, Faster R-CNN and Cascade R-CNN, achieved acceptable precision (76.2% and 79.0%) and recall (79.5% and 82.4%). However, their core mAP metrics were significantly lower than those of the lightweight one-stage models. This indicates that two-stage detectors struggle with the localization and classification of dense, morphologically similar, and small-scale maize seedlings and weeds in complex field environments. More critically, their computational complexity is extremely high (FLOPs of 95.2 G and 67.4 G; parameters of 41.3 M and 69.2 M), resulting in inference speeds of only 19.1 FPS and 15.3 FPS, respectively, which fail to meet the strict requirements for real-time detection and edge deployment in agricultural scenarios.

Performance analysis of the one-stage detectors: Among the one-stage detectors, RTMDet performed poorly on this task. Its recall was only 78.1%, and its mAP@0.5 was merely 75.7%. This suggests difficulties in handling targets with highly variable morphologies and complex backgrounds. The YOLO series performed excellently; YOLOv5n demonstrated advantages in lightweight design and speed (FLOPs of 7.1 G, 1.9 M parameters, and 62.3 FPS); however, its precision (79.3%) and mAP@0.5 (80.6%) were relatively low. The YOLOv8n baseline achieved a good balance between accuracy and efficiency, with an mAP@0.5 of 83.2%, which was significantly higher than that of the aforementioned comparison models, while maintaining low complexity and high speed (8.1 G FLOPs, 3.1 M parameters, and 58.2 FPS), justifying its selection as the baseline for this study.

Analysis of the advantages of the proposed model: The proposed DCC-YOLOv8n model achieved the best overall performance among all the compared models. In terms of detection accuracy, the precision, recall, mAP@0.5, and mAP@0.5:0.95 reached 90.1%, 95.5%, 93.7%, and 73.9%, respectively. The superior performance of the proposed model fully validates the effectiveness of the incorporated improvements, including dynamic convolution, context guidance, and content-aware upsampling. Regarding efficiency, DCC-YOLOv8n reduced the computational complexity and parameters by approximately one order of magnitude compared with large mainstream models such as Faster R-CNN. With 11.2 G FLOPs and 4.0 M parameters, the model achieved a lightweight design. Although its inference speed of 41.3 FPS is lower than that of extremely lightweight models such as YOLOv5n, it remains significantly faster than two-stage detectors and fully satisfies the requirements for real-time field processing. This demonstrates that by introducing specialized modules for complex farmland scenarios, the proposed model successfully realizes the goal of significantly enhancing the accuracy while controlling the complexity. It achieves an optimal balance between accuracy, lightweight architecture, and speed, outperforming mainstream methods and providing a solid technical foundation for high-precision real-time weed recognition on resource-constrained edge-computing devices.

#### 3.2.2 Visualization analysis of model detection results

##### 3.2.2.1 Adverse condition testing

**Figure 13:**
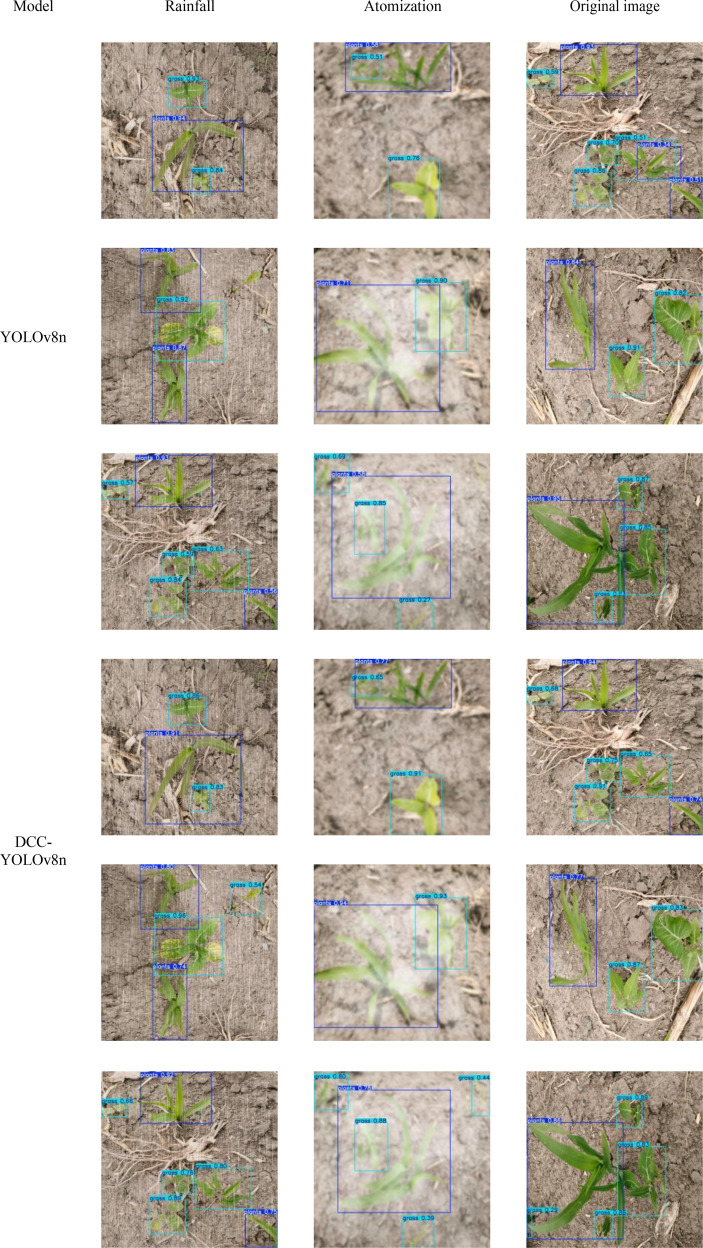
Visual comparison of detection results. Dark blue boxes denote the model-predicted regions for maize seedlings, while light blue boxes indicate the predicted regions for weeds. The numerical values above the boxes represent the model’s confidence scores for the respective predictions.

To demonstrate the performance improvement of the enhanced model intuitively, this study selected typical complex field scenarios, including foggy weather, rainy weather, dense target distributions, and the presence of small target weeds, to conduct a visual comparison of the detection effects between the baseline YOLOv8n and the proposed DCC-YOLOv8n model. A visualization of the detection results is shown in **Figure 13**.

Robustness in complex backgrounds: Under low-visibility conditions such as foggy weather, the original YOLOv8n model exhibited significant missed detections. In contrast, the DCC-YOLOv8n model, through its CG module, effectively fused multiscale contextual information, enhancing its global perception of targets and significantly reducing missed detections, thereby demonstrating its superior environmental adaptability.

Detection capability for small-scale targets: In scenarios involving small or occluded weeds, the original model struggled to identify inconspicuous or partially obscured targets. By leveraging the CARAFE upsampling module, the ability of the DCC-YOLOv8n model to retain and reconstruct detailed features was enhanced. Combined with multiscale feature fusion, this improved the detection accuracy for small and occluded targets.

Localization and classification confidence: Across all compared scenarios, the bounding boxes generated by the DCC-YOLOv8n model fitted the target contours more closely, and the predicted confidence scores were generally higher. This indicates that the improved feature representation mechanism not only bolsters the classification certainty but also enhances the regression accuracy of the bounding boxes.

In summary, the visualization results indicate that the DCC-YOLOv8n model outperforms the original YOLOv8n in terms of target recall, small-target detection, and localization accuracy across various typical complex field scenarios. These findings are consistent with the quantitative analysis and further validate the effectiveness of the proposed improvement strategies.

##### 3.2.2.2 Typical failure cases

**Figure 14:**
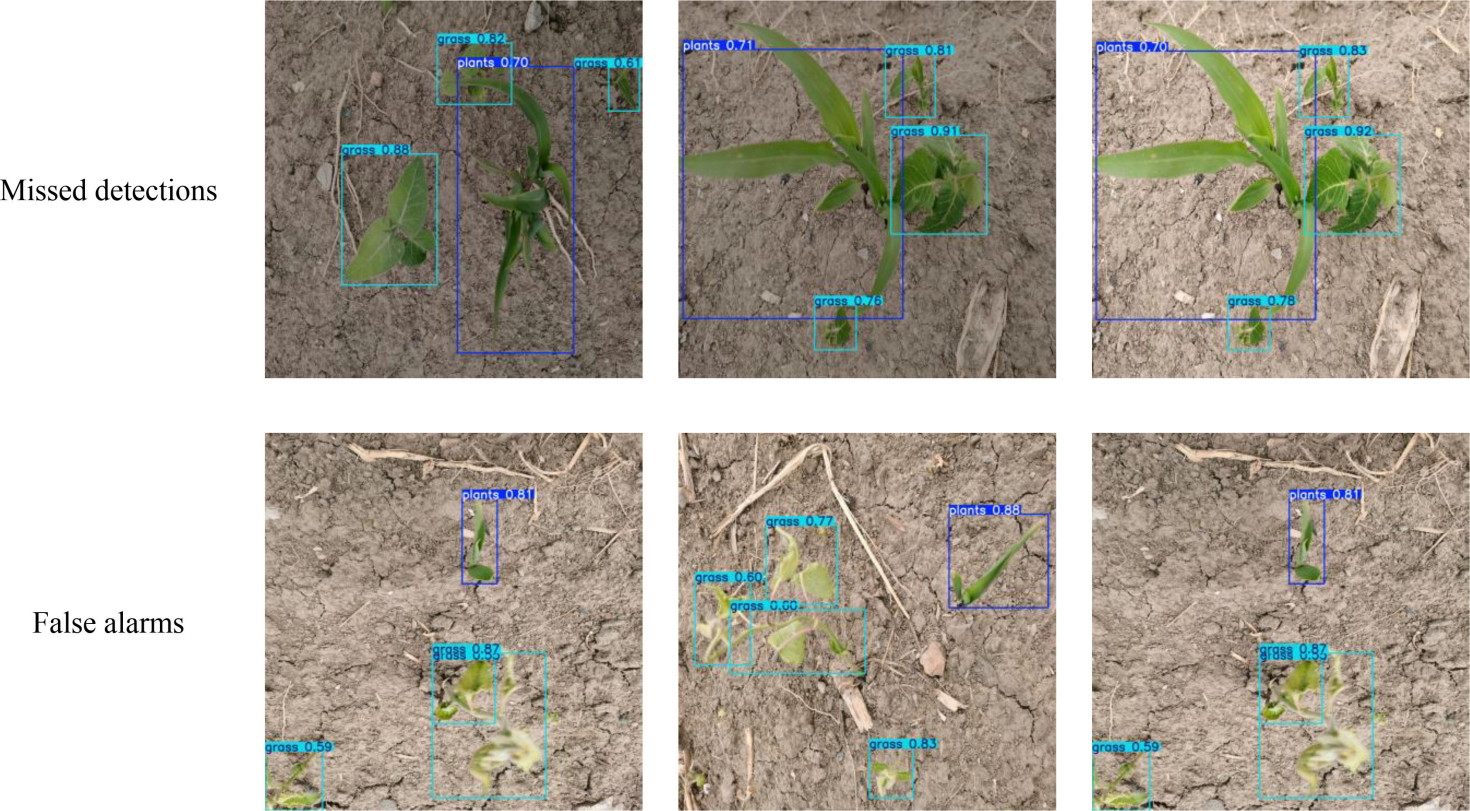
Visualization of representative failure cases.

Analysis of typical failure cases in the DCC-YOLOv8n detection results reveals two predominant error patterns. False negatives​ primarily occur in scenarios involving multiple maize seedlings within a single planting hole; when two adjacent seedlings are in close proximity, the model tends to merge them into a single target, yielding only one bounding box. Conversely, false positives are mainly observed with large weed instances, where the model erroneously fragments a single plant into multiple distinct targets, generating several redundant bounding boxes. These two representative failure modes directly degrade the overall recognition accuracy. Typical failure cases are illustrated in **Figure 14**.

#### 3.2.3 Real-time deployment verification on edge devices

To ensure comparability and fairness of the experiments, the same validation set was used to evaluate the performance of the maize seedling and weed recognition system deployed on the NVIDIA Jetson Nano. The test results are listed in **Table 10**.

**Table 10:** Test results.

Model	Precision(%)	Recall(%)	mAP@0.5(%)	FPS
YOLOv8n(on PC)	79.5	84.4	83.2	58.2
DCC-YOLOv8n(on PC)	90.1	95.5	93.7	41.3
YOLOv8n(on Nano)	77.6	81.2	80.7	25.7
DCC-YOLOv8n(on Nano)	88.3	91.6	90.8	18.6

Based on the results in **Table 10**, a comparative analysis of model performance was conducted between the NVIDIA Jetson Nano edge computing platform and the PC terminal across two dimensions: accuracy and efficiency. Regarding detection accuracy, the DCC-YOLOv8n model comprehensively outperformed the baseline YOLOv8n on both the PC and Jetson Nano, verifying the effectiveness of the three improvement strategies: DC, CG, and CARAFE. The model maintained its accuracy advantage on the edge device, consistent with performance on the PC; specifically, it achieved an mAP@0.5 of 90.8% on the Jetson Nano, representing a 10.1% improvement over the baseline on the same platform, which demonstrates robust cross-platform performance. Although the transition from PC to edge deployment introduced a slight accuracy loss due to computational power constraints and FP16 quantization (e.g., mAP@0.5 decreased from 93.7% to 90.8%), the reduction remained within a reasonable range, and the improved model maintained a significant performance edge on the device.

In terms of inference efficiency, the structural enhancements of DCC-YOLOv8n led to a 29.0% increase in parameters and a 38.3% increase in computational load compared with the baseline, resulting in a decrease in inference speed. On the PC, the FPS dropped from 58.2 to 41.3; on the Jetson Nano, FPS decreased from 25.7 to 18.6. This reflects a design tradeoff in which moderate computational overhead is exchanged for significant accuracy gains. The constraints of edge computing power were evident, as the inference speed of the model on the Jetson Nano was approximately 45% of its speed on the PC. Ultimately, DCC-YOLOv8n achieved 18.6 FPS on the Jetson Nano, satisfying the real-time requirements for agricultural mobile platforms (Islam et al., 2025) and achieving an effective balance between accuracy, complexity, and edge real-time performance.

## 4 Discussion

In this study, addressing the task of maize seedling and weed recognition in complex field environments, a lightweight detection model, DCC-YOLOv8n, was constructed based on the YOLOv8n architecture. Simultaneous improvements in both detection accuracy and efficiency were achieved through the synergistic optimization of the data augmentation and network structure.

A comparative analysis with other improvement strategies indicates that most existing studies optimized a single dimension, such as enhancing backbone network structures (Ding et al., 2024; Liu et al., 2024; Zheng et al., 2024), improving feature fusion modules (He et al., 2024; Ren et al., 2024), and exploring novel loss functions (Guo et al., 2023). In contrast, this study proposes a multipath collaborative optimization approach. The network was systematically enhanced across three dimensions—feature representation, contextual understanding, and feature detail reconstruction—by incorporating DC, CG, and CARAFE modules, respectively. This design comprehensively bolsters the capabilities of the model for feature discrimination, environmental robustness, and small target detection.

The proposed improvement strategies exhibit clear systematicity and synergy: the DC module enhances feature representation through input-adaptive convolution kernel aggregation, helping to distinguish morphologically similar targets; the CG module improves target identification in complex backgrounds by fusing multiscale contextual information; and the CARAFE module reduces detail loss during upsampling through content-aware feature reassembly, refining the detection of small-scale weeds. These three modules operate collaboratively at the feature extraction, context fusion, and feature reconstruction levels, forming an optimization chain that covers the key stages of object detection.

In summary, the DCC-YOLOv8n model provides a balanced solution that integrates a high-precision, lightweight architecture and strong robustness. The experimental results demonstrate that the model outperforms the YOLOv8n baseline and mainstream comparison models in terms of mAP, computational complexity, and inference speed, effectively addressing the challenges of complex field environments.

Despite the significant performance gains, scope for optimization remains. First, the dataset coverage of different growth stages, extreme environmental conditions, and diverse weed species is not exhaustive. Second, the deployment efficiency and stability of the model on practical edge devices require long-term validation. Third, a formal quantitative evaluation stratified by object scale remains to be conducted. Future work will focus on the following areas: expanding the dataset to enhance generalization, further optimizing the model for deployment on various hardware platforms, such as Jetson Nano, to promote practical applications, and exploring fine-grained farmland scene understanding methods that incorporate temporal information or multitask learning. Additionally, detailed quantitative assessments across fine-grained size categories will be conducted to further validate the model’s capability in detecting sparse and diminutive weed instances in complex field scenarios.

## 5 Conclusion

This study proposes a lightweight maize seedling and weed recognition model, DCC-YOLOv8n, designed for complex farmland environments. Comparative experiments with mainstream models, including Faster R-CNN, Cascade R-CNN, RTMDet, and YOLOv5n, demonstrated that DCC-YOLOv8n achieves superior performance across key evaluation metrics, such as precision, recall, and mAP, on the PGD dataset. Simultaneously, the model achieves an optimal balance between the parameter count, computational complexity, and inference speed, characterized by its lightweight nature and high accuracy, making it highly suitable for real-time field recognition tasks in resource-constrained environments.

Based on this model, a recognition system was successfully deployed on an NVIDIA Jetson Nano edge computing device. Offline validation trials indicated that the system maintains excellent recognition accuracy and real-time processing capabilities across various scenarios, including complex weather conditions, variable lighting, occlusion, and small targets. The system effectively supports precision weeding and variable-rate agricultural machinery operations, providing reliable technical support for the intelligence and automation of agricultural production.

Overall, the maize seedling and weed recognition system constructed in this study achieves efficient and precise object detection in complex farmland scenarios and verifies the feasibility of practical deployment on edge devices. This work not only provides a new perspective for designing lightweight agricultural vision models but also offers a viable technical path for the practical application and sustainable development of smart agriculture technologies, demonstrating significant application prospects and promotional value.

## Data Availability

The original contributions presented in the study are included in the article/supplementary material. Further inquiries can be directed to the corresponding author.
